# Supplementary material for: Edible Insect Meals as Bioactive Ingredients in Sustainable Snack Bars
Source: Foods. 2025 Feb 18;14(4):702. doi: 10.3390/foods14040702 (PMC11854822; doi:10.3390/foods14040702)
Supplement: Supplementary file 1 [file foods-14-00702-s001.zip › foods-3475013-supplementary.pdf]

Table S1. Interviewees of different age groups and distinguished by gender, educational qualification and place of residence.

		Age group (years)														
		16/17	18/20	21/25	26/30	31/35	36/40	41/45	46/50	51/55	56/60	61/65	66/70	71/75	76/80	81/85
	**Number interviewed**	**20**	**30**	**50**	**50**	**50**	**50**	**50**	**50**	**50**	**50**	**50**	**50**	**50**	**50**	**50**
**Gender**	Male	9	12	25	27	26	20	23	28	25	25	24	21	25	26	23
	Female	11	18	25	23	24	30	27	22	25	25	26	29	25	24	27
**Qualification**	Degree	0	0	36	41	45	47	46	46	46	46	40	35	35	35	35
	High school	0	21	12	9	5	2	3	3	3	3	2	5	5	5	5
	Middle school	20	9	2	0	0	1	2	2	2	2	8	10	10	10	10
**Residence**	**city** > 50,000 residents	6	10	13	14	13	14	14	15	14	13	14	14	13	15	14
	10,000 < **city** < 50,000 residents	7	9	15	16	17	20	18	23	21	16	10	15	18	17	15
	**city** < 10,000 residents	7	11	22	20	20	16	18	12	15	21	26	21	19	18	21

Table S2. Sensory analysis form

Panelist	SAMPLE CODE

ATRRIBUTES	CRITERIA AND EVALUATION								
Appearance	Overall appearance of the bar, including shape, surface perception, presence of visible insect fragments and uniformity, was evaluated according to the following criteria: - Is the shape consistent and well modelled? - Are visible insect components present and do they affect acceptability?								
	**1**	**2**	**3**	**4**	**5**	**6**	**7**	**8**	**9**
Color	Appropriateness in relation to consumer expectations, expressed as a summative judgement with respect to the following criteria: - Is the color uniform and natural? - Does the color correspond to the expectations of a protein/energy bar? - Are there any discolorations or unattractive shades?								
Flavor	The overall taste profile, including sweetness, bitterness, umami and potential off-flavours, expressed as the summative judgement of the following criteria: - Is the taste balanced and pleasant? - Are there dominant flavours - Does the bar have a desirable level of sweetness and seasoning?								
	**1**	**2**	**3**	**4**	**5**	**6**	**7**	**8**	**9**
Aroma	The intensity and pleasantness of the odor of the bar, was reported as a summative judgement with respect to the following questions: - Is the aroma inviting or neutral? - Are there strong or unpleasant odors related to insects? - Is the aroma consistent with that of conventional protein bars?								
	**1**	**2**	**3**	**4**	**5**	**6**	**7**	**8**	**9**
Texture	As mouthfeel and structural integrity of the bar, including hardness, chewability and softness, is expressed as a summative judgement of the following criteria: - Is the texture soft, firm or too hard? - Does it crumble, stick to the teeth or is it grainy? - Is chewability acceptable?								
	**1**	**2**	**3**	**4**	**5**	**6**	**7**	**8**	**9**
Stickiness	Degree of adherence of the bar to the fingers, and to the oral cavity during consumption, is expressed as a summative judgement of the following criteria: - Is it excessively sticky when handled? - Does the bar leave a sticky residue in the mouth? - Does the level of stickiness affect overall acceptability?								
	**1**	**2**	**3**	**4**	**5**	**6**	**7**	**8**	**9**
Aftertaste	Residual taste that remains in the mouth after swallowing, is expressed as a summative judgement of the following criteria: - Is the aftertaste pleasant, neutral or undesirable? - Is the aftertaste long-lasting? -Does the aftertaste encourage or discourage further consumption?								
	**1**	**2**	**3**	**4**	**5**	**6**	**7**	**8**	**9**

Overall liking	Overall acceptability of the bar based on all sensory attributes combined, is expressed as a summative judgement of the following criteria: - Would the consumer consume the bar again? - Is the bar as enjoyable as or better than conventional protein bars?								
	**1**	**2**	**3**	**4**	**5**	**6**	**7**	**8**	**9**

Scale Score: 1, Dislike Extremely; 2, Dislike Very Much; 3, Dislike Moderately; 4, Dislike Slightly; 5, Neither Like nor Dislike; 6, Like Slightly; 7, Like Moderately; 8, Like Very Much; 9, Like Extremely

Document S1. Online Anonymous Survey Consent

Hello,
I'm Patrizio Tremonte, a professor at University of Molise. I am conducting an online survey to learn about familiarity with insect-based foods, to understand the propensity to consume them. To participate you must be between 16 and 85 years old.

The following survey includes questions about:
- the size of the city/village of residence
- gender, -
- educational qualifications,
- the level of knowledge of insect-based foods;
- propensity to consume insect-based foods;
- the preferred type of insect-based foods, if any;
- the expected claims in possible insect-based food

- Your participation in the survey is voluntary and data will be collected anonymously through the completion of online questionnaires. The objective of this survey is to be exploring nature and your contribution will be of great help in increasing knowledge and information on the area under study (consumption of insect foods). There are no disadvantages, risks or dangers involved in taking part in the survey.
- The information collected by this questionnaire will be used only for research purposes in scientific projects conducted at the Department of Agriculture, Environment and Food of the University of Molise without any involvement of private companies.
- All data will be collected and processed anonymously, with no identification of participants and no distinguishing features that can be traced back to the identity of the participants. Only the person responsible for the research and authorized personnel will have access to the material collected and it will not be possible to identify participants in any way. The data collected will only be used for this investigation and will not be passed on to anyone outside the research team. All data collected will only be analyzed in aggregate form and the results of the study may be reported in scientific publications. It will not be possible to identify any research participants among the results when they are published.
- In accordance with Legislative Decree 196/2003, "Code on the Protection of Personal Data" and GDPR (EU Regulation 679/2016) "European Regulation on the Protection of Personal Data," available at https://www.lavoro.gov.it/Documents/informativa_GDPR.pdf, each respondent is asked for their consent to the participation and processing of data of which this information is an integral part.
- - Pursuant to Articles 15 to 22 of the GDPR and Legislative Decree 101/2018, one may withdraw from the consent provided at any time and without a justified reason by sending a request via email to the research manager.
- The questionnaire consists of questions whose answers can be guided or refer to scoring scales from 1 (not at all) to 10 (very much)
- To obtain further information or clarification regarding the survey, as well as to withdraw consent, it is possible to contact the research contact persons Prof. Patrizio Tremonte at the e-mail address: tremonte@unimol.it and Dr. Silvia Jane Lombardi at the e-mail address: silvia.lombardi@unimol.it
- At the end of reading this information, if you wish to participate in the research, before proceeding to fill out the questionnaire, you must provide your personal consent to participate in the research and data processing.

Full Name:____ Date____________________

□ I HEREBY DECLARE that I have read this notice and have received the information necessary to understand the objectives and methods of participation in the survey

(Signature) ____________________________________

□ I GIVE MY CONSENT to participate in the survey under the conditions and in the manner indicated in the information notice

(Signature) ____________________________________

□ I HEREBY GIVE MY CONSENT to the processing of personal data (art. 13 of D.L. n.196/2003 and art. 13 of GDPR n. 679/2016 EU Regulation)

(Signature)____________________________________

Document S2. Online Anonymous Survey Consent - PARENTAL PERMISSION - Minor Assent (ages 16- <18 years)

Dear Parent,

I'm Patrizio Tremonte, a professor at Universyty of Molise.
I am conducting an online survey to learn about familiarity with insect-based foods, to understand the propensity to consume them. To participate you must be between 16 and 85 years old.

So, your child is eligible to participate in this research.

If your child participates in this research, they will be asked to:

- the size of the city/village of residence
- gender
- educational qualifications
- the level of knowledge of insect-based foods
- propensity to consume insect-based foods
- the preferred type of insect-based foods, if any
- the expected claims in possible insect-based food

All questions are optional: your child can skip any part of this research that they do not wish to complete. They can stop participating at any time.
Your child's participation in this study is voluntary. If you choose not to have your child participate or choose to withdraw your child from the study, there will be no penalty. If your child chooses not to participate or to withdraw from the study, he or she may do so at any time without penalty. The results of the research study will be published, but your child's name will not be used. To maintain confidentiality of your child's records, I will collect and process all data anonymously, with no identification of participants and no distinguishing features that can be traced back to the identity of the participants. Only the person responsible for the research and authorised personnel will have access to the material collected and it will not be possible to identify participants in any way.
This study will help us learn more about food security
I do not foresee any problems from this study, but your child may be experimenting with a food novelty
If you feel pressured to allow your child to participate at any time, or if you have any questions concerning this study, please call me on: + 39 0874 404849

If you have any questions regarding your child's rights as a research participant, you may email me tremonte@unimol.it or Dr Silvia Jane Lombardi silvia.lombardi@unimol.it.

By signing below, you are allowing your child to participate in this study. Please keep one copy of this form for your records.

Child's Name__________________________

Parent's Name___________________ Parent's Signature___________________ Date_________________

Document S3. Consent Sensory Evaluations

Dear Sensory Panelist,

People 18 years of age and older are invited to participate in a research study to evaluate sensorial attributes of insect-based snack bars. This project is being conducted by Prof. Patrizio Tremonte from Department of Agricultural, Environmental and Food Sciences at University of Molise

PURPOSE OF THE STUDY: The sensory analyses are conducted by trained volunteers and are part of a larger study to identify user-friendly solutions based on the use of insect meals that could improve the microbiological safety and consumer acceptability of insect-based foods.

DETAILS:

- Dates: Each volunteer will participate in 10 training sessions and then perform a triple sensory analysis on two batches of snack bars. Conventional ingredients, by-products of different food chains and insect flours compose the bars for sensory analysis. Each panelist conducts a threefold sensory analysis on the bar using a 9-point hedonic scale.
- Time Commitment: Each volunteer panelist participates in 10 training sessions and 3 sensory analysis sessions. Each session's duration is 60 minutes.
- Information Location: Sensory analyses are conducted at the Department's facilities

EXPLANATION OF PROCEDURES: Panelist conducts a threefold sensory analysis on the bar using a 9-point hedonic scale to assess appearance, color, aroma, flavor, texture, stickiness, aftertaste and overall liking.

VOLUNTARITY: participation is voluntary, and the panelist may withdraw at any time without penalty.

Your help will be greatly appreciated in making this study meaningful.

PRIVACY AND CONFIDENTIALITY: All information obtained during the testing procedures will remain confidential and will not be visible to other panelists or people not associated with this study. The identity of the panelist will not be revealed in the results of the experiment. Only comparisons will be made and reported in summary form. The data will only be accessible to the researchers of the study and may only be used for the purpose of scientific publications in journals intended for the scientific community.

POTENTIAL BENEFITS AND RISKS: Participation in the study does not result in any direct benefits but does produce useful new knowledge in the field of food security.

CONTACT INFORMATION: If you have any questions about this project, please contact via email: tremonte@unimol.it or silvia.lombardi@unimol.it.

Documentation of Informed Consent:

You are freely making the decision to be in this research study. By signing this form, you indicate that:

1. You are 18 years of age or older,
2. you have read and understood this consent form,
3. you have had your questions answered,
4. you have decided to be in the study.

You will be given a copy of this consent form to keep.

_______________________________________ ____________________

Your Signature Date
